# High Efficiency Uranium(VI) Removal from Wastewater by Strong Alkaline Ion Exchange Fiber: Effect and Characteristic

**DOI:** 10.3390/polym15020279

**Published:** 2023-01-05

**Authors:** Shiping Zhou, Faqin Dong, Yilin Qin

**Affiliations:** 1 School of Environment and Resource, Southwest University of Science and Technology, Mianyang 621010, China; 2 Key Laboratory of Solid Waste Treatment and Resource Recycle, Ministry of Education, Mianyang 621010, China; 3 Fundamental Science on Nuclear Wastes and Environmental Safety Laboratory, Mianyang 621010, China

**Keywords:** strong alkaline ion exchange fiber, uranium(VI), deep purification

## Abstract

In this study, we analyzed the removal efficiency of uranium(U(VI)) in wastewater at relatively low concentrations using strong alkaline ion exchange fiber (SAIEF). Static tests showed that the strong alkali fibers can purify U(VI) containing wastewater in a concentration range of 20–100 mg L^−1^ with an optimal pH of 10.5 and contact time of 15–30 min. Adsorption and desorption cycling tests indicated that, adsorbed uranium is easily desorbed by 0.1 mol L^−1^ HCl, and the fiber still maintained the original adsorption efficiency after eight cycles. According to dynamic penetration test results, the SAIEF saturation adsorption capacity was 423.9 mg g^−1^, and the effluent concentration of uranium through two series columns was less than 0.05 mg L^−1^, reaching the national standard for non-receiving water (GB23727-2009) SEM-EDS and FTIR analysis revealed that the functional group of SAIEF is CH_2_N^+^(CH_3_)_3_Cl^−^. Addotionally, the major forms of fiber exchange adsorption are (UO_2_)_2_CO_3_(OH)_3_^−^, UO_2_(CO)_3_^4−^ and UO_2_(OH)_3_^−^. The results indicate that the SAIEF is an excellent material for uranium removal.

## 1. Introduction

As a key element in the nuclear fuel cycle, uranium has been widely used in nuclear, isotope test and production reactors. However, nuclear fuel utilization produces significant radioactive effluent owing to its radioactivity and toxicity, posing a huge threat to human health and the environment [1,2,3]. Therefore, efficient treatment of radioactive uranium-containing wastewater is a major sustainable development problem in the nuclear fuel cycle industry that needs to be addressed.

Typical treatment techniques of uranium-containing wastewater include chemical methods, such as chemical precipitation [4], electrolysis [5], adsorption [6,7,8,9,10,11,12,13], photocatalytic reduction [14,15] and ion exchange [16], and physical-chemical methods, such as evaporation enrichment method [17], extraction [18,19], ion flotation [20] and membrane filtration [21,22,23].

Among the technologies, adsorption, which is inexpensive, highly selective, and simple to operate, is considered one of the most effective technologies to treat U(VI) containing wastewater. K. Sakr et al. [24] used clay to remove the uranium from nuclear effluent using regenerated bleaching earth immersed in β-naphthol. Li et al. [25] studied the effect of different molecular chain conformations on the uranium adsorption ability of amidoxime adsorbents. Huang et al. [26] utilized hyperbranched polyethyleneimine-functionalized PAN (PAN-HPEI) fibers and confirmed that the total process was accomplished within 30 min with an equilibrium adsorption capacity of 465 mg g^−1^ and pH of 6.0.

Among all the adsorbents, ion exchange fiber, a new type of synthetic fiber, has lately been used in mental adsorption with the special advantages such as higher adsorption/desorption rate and a stronger absorption capacity owing to the shorter transport distance of ions and the number of functional groups present along the fiber for industrialization [27,28]. Zhou et al. [29] studied the mental-adsorption ability of the strong acidic cation exchange fiber combined with the sulfonic acid groups. The saturation adsorption capacity of Pb^2+^ and Cd^2+^ was 206.6 mg g^−1^ and 105.5 mg g^−1^, respectively. Additionally, the time to reach equilibrium was only 5–10 min, and the fiber was easy to recycle. Zeng et al. [30,31] also studied the adsorption of Cu^2+^, Co^2+^, and Ni^2+^ by modified fiber, indicating the adsorption potential of ion exchange fibers. However, according to available data, deep purification of high-concentration radioactive uranium-containing wastewater using ion exchange fiber has been rarely studied.

In this study, the removal properties and related mechanism of strong alkaline ion exchange fiber (SAIEF), a new type of fabric absorption and separation material, for high-concentration uranium were studied. The conditions affecting U(VI) capture by SAIEF were investigated, including pH, contact time, initial uranium concentration, and fiber dosage. Through the kinetics, adsorption isotherm curve and thermodynamics, the possible interaction between U(VI) and SAIEF was proposed. The study can provide a valuable theoretical basis for the research of potential applications for uranium-containing wastewater.

## 2. Material and Methods

### 2.1. Materials and Reagents

**Figure 1:**
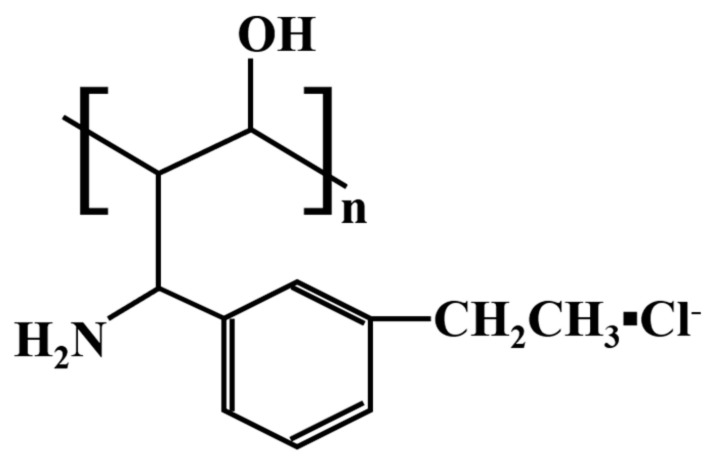
Schematic of SAIEF structure.

Strong alkaline anion exchange fiber (length of 12 mm, and diameter of 57–75 μm) were purchased from the National Engineering and Technology Research Centre for Municipal Wastewater Treatment and Reuse (Mianyang, China). SAIEF was a modified polymer fiber, which was prepared by radiation grafting, chloromethylation and amination reactions of polypropylene as fundamental materials [32], the structure of SAIEF is shown in Figure 1.

NaCl, concentrated HCl (37 wt%) and NaOH were purchased from Aladdin Co., Ltd. (Shanghai, China). UO_2_(NO_3_)_2_·6H_2_O was supplied by Chengdu Jinshan Co., Ltd. (Chengdu, China). Na_2_CO_3_ was supplied by Chengdu Kelong Chemical Co., Ltd. (Chengdu, China). In this study all the reagents were analytical grade and used without further purification. Deionized (DI) water was used in all experiments.

The uranium-containing wastewater used in this study was simulated wastewater configured in a laboratory. Briefly, 2.1092 g uranyl nitrate [UO_2_(NO_3_)_2_·6H_2_O] was dissolved in water. After adding 10 mL of dilute nitric acid, the solution was diluted to 1000 mL with DI water. A stock solution of U(VI) (1.0 g L^−1^) was prepared and diluted to the different initial concentrations for standby application. The initial pH of the simulated uranium containing wastewater was adjusted by 0.1 mol L^−1^ HCl and 10 g L^−1^ Na_2_CO_3_.

### 2.2. Characterization Techniques

To determine the surface morphology and element compositions of the fibers scanning electron microscopy (SEM, Zeiss, Oberkochen, Germany) and energy dispersive X-ray spectrometry (EDS) were used in this work. The changes in functional groups on the fibers were characterized using Fourier transform infrared spectrometer (FT-IR, Spectrum One, PE Instrument Company); The concentration of metal ions in the solution was determined using inductively coupled plasma optical emission spectrometer (ICP-MS, Agilent 7700×, Agilent, Santa Clara, CA, USA).

### 2.3. Methods

#### 2.3.1. Preparation of SAIEF

Briefly, 8 g of strong alkaline anion exchange fiber (SAIEF) was washed with pure water, and 400 mL 1.0 mol L^−1^ HCl solution was then added. After stirring at 30 °C for 12 h, the fibers were rinsed to neutral with DI water and squeezed. Subsequently, 400 mL of 1.0 mol L^−1^ NaOH solution was added with continuous stirring at 30 °C. After 12 h, the fibers were washed to neutral with ultra-pure water. Finally, the fibers were taken out and squeezed to dry.

#### 2.3.2. Static Adsorption Experiments

Static adsorption experiments were conducted to analyze the best conditions for fibers. Uranium solution was diluted to different concentrations (5 mg L^−1^, 10 mg L^−1^, 20 mg L^−1^, 50 mg L^−1^, 100 mg L^−1^), and the pH was adjusted to a specific value in batches. The fibers were added into predetermined U(VI) solution (100 mL) while stirring. Through different contact times, the concentration of uranium was determined via Agilent 7700× inductively coupled plasma optical emission spectrometer. The effects of pH, dosage of fibers, initial uranium concentration, and contact time on the uranium adsorption efficiency were investigated.

After repeating the optimal condition test, different desorption agents were used for adsorbed uranium desorption test. The best desorption agent was selected, with which, desorption and reabsorption cycling tests were conducted.

#### 2.3.3. Dynamic Adsorption Experiments

To investigate the deep purification of uranium wastewater, the dynamic column adsorption behavior of SAIEF was conducted using the column filled with fibers and a two-grade serial dynamic adsorption experiment.

### 2.4. Calculation

U(VI) concentration was determined by WGJ-III trace uranium analyzer. The standard sample was provided by Hangzhou Daji Photoelectric Instrument Co., Ltd. Adsorption capacity of SAIEF was calculated using the following equation:

(1) $$R=\frac{C_{0}-C_{t}}{C_{0}}\times 100\%$$

(2) $$q=\frac{\left(C_{0}-C_{t}\right)V}{m}$$

where *R* is the adsorption rate (%); *q* is the adsorption capacity (mg·g^−1^); *C*_0_ and *C*_t_ are U(VI) concentration of before and after adsorption, respectively (mg·L^−1^); *V*_0_ and *V*_t_ are total solution volume (L); *m* is the weight of SAIEF (g).

## 3. Results and Discussion

### 3.1. Characterization of SAIEF

#### 3.1.1. SEM/EDS Analysis of SAIEF

**Figure 2:**
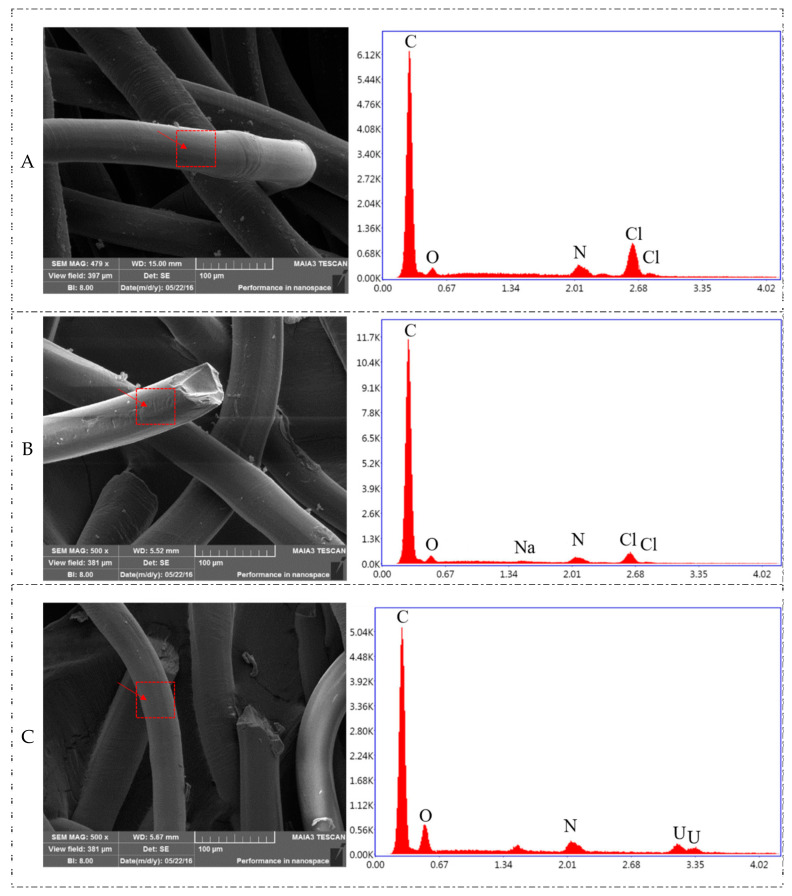
The SEM and EDS diagrams ((**A**): original fibers; (**B**): prepared fibers; (**C**): adsorption of uranium fibers).

The SEM/EDS analyses of the changes in surface morphology and chemical constituents of original, as-prepared, and after-adsorbed fibers are shown in Figure 2. From the EDS analysis, the existence of C, Cl, N and O elements in the fibers could be confirmed and the fibers were solid bundles with a diameter of 55–75 μm. After preparation, the main elemental composition on the surface of the strong alkaline ion exchange fibers was barely changed. A small amount of Na and O elements slightly increased owing to the use of NaOH solution in the preparation. Furthermore, the characteristic peak of the Cl element is approximately 2.68 KeV.

In contrast to the EDS analysis of prepared SAIEF, the characteristic peak of the Cl element disappeared after adsorption. The characteristic peak of U element was identified at 3.35 KeV, and the characteristic peak of the O element was enhanced. It is inferred that the functional group of the SAIEF contains the Cl element, and the form of the fiber adsorbing uranium is at least a composite anion formed by U and O elements. 

#### 3.1.2. FTIR Analysis

**Figure 3:**
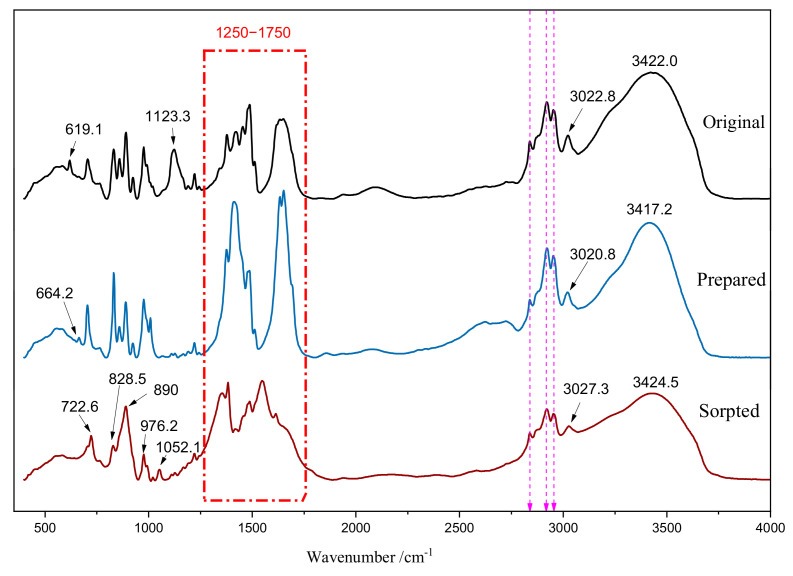
FTIR spectra of SAIEF.

Figure 3 presents the FTIR spectra of original, as-prepared, and after-adsorbed ion exchange fibers (4000–400 cm^−1^). The FTIR spectra of original fibers exhibited bonds at 3422 cm^−1^, 2963 cm^−1^, 1453 cm^−1^ and 1377 cm^−1^ corresponding to the associative -OH vibration or N-H stretching vibration, C-H stretching vibration of -CH_2_CH_3_, and -CH_2_CH_3_ bending vibration, respectively, indicating that SAIEF has a polypropylene structure [33,34,35]. The strong peak at 1649 cm^−1^ and 1488 cm^−1^ can be attributed to the benzene ring, and the peak at 1649 cm^−1^ can be attributed to the bending C-N-H. The bands at approximately 1221–1019 cm^−1^ and 800–600 cm^−1^ corresponded to the N-C stretching vibration and C-Cl stretching vibration respectively. Moreover, the peak position fluctuated with different apparent substitutions, indicating that the fiber has a C-Cl bond, which corroborates with the EDS results.

After preparation, the number of the peaks decreased in the range of 1750–1250 cm^−1^, the remaining peaks were almost the characteristic peaks of polypropylene skeleton, benzene ring and N-C stretching vibration. After preparation a new absorption peak of C=C appeared at 1633 cm^−1^, indicating that preparation can optimize the fiber. The peaks in the halogen region between 800 cm^−1^ and 600 cm^−1^ significantly changed, indicating that the pretreatment had a significant effect on the distribution of active groups on the fibers.

After saturation adsorption, the FTIR spectra evidently changed. The peak of the polypropylene skeleton was generally constant, implying that the U(VI) adsorption had little effect on the structure of polypropylene structure. The adsorption peaks at approximately 2950 cm^−1^, 2920 cm^−1^, and 1412 cm^−1^ became weak. The peaks at 1649 cm^−1^ and 1633 cm^−1^ disappeared, however, new adsorption peaks appeared at 1613 cm^−1^, 1548 cm^−1^, and 1412 cm^−1^. The results indicate that benzene ring, C=C, -CH_2_CH_3_, C-H, C-N-H and other groups were involved in the exchange adsorption behavior of U(VI). C-H out-of-plane bending vibration was located at 722.6 cm^−1^. The peak reappeared at 890 cm^−1^ and was significantly enhanced, which may be the characteristic absorption peak of UO_2_^2+^ [36,37]. This confirms the reaction between ion-exchange fibers and uranium-containing ions in U(VI)-containing wastewater.

### 3.2. U(VI) Adsorption Properties 

#### 3.2.1. Effect of pH

**Figure 4:**
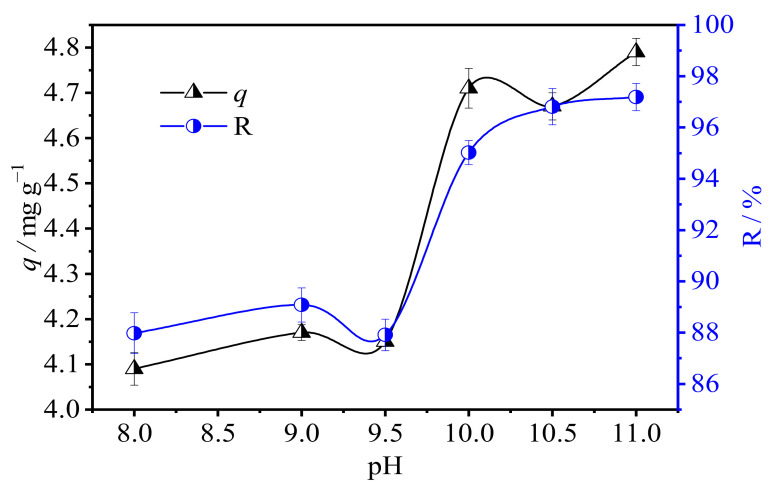
Effect of pH on the adsorption of U(VI) by SAIEF (C_0_ = 50 mg L^−1^, solid–liquid ratio = 0.15 g L^−1^, t = 60 min).

pH typically often has a great influence on the form of ions in solution, which is considered a significant factor during the U(VI) uranium by ion exchange fiber [38]. Due to the strong alkali ion exchange fiber (SAIEF), the pH in this study was set at a range of 8–10. The initial concentration of uranium solution was 50 mg L^−1^ (C_0_), and the solid-liquid ratio was 1.5 g 100 mL^−1^. The effect of pH on the U(VI) adsorption capacities at the contact time of 1 h is shown in Figure 4. 

**Figure 5:**
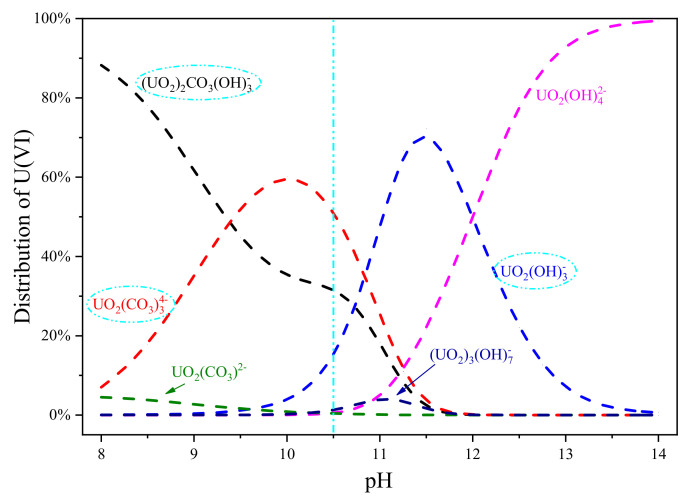
Distribution of uranium ions at pH 8–14 in this study system.

The initial U(VI) adsorption rate was approximately 88%, and with an increasing pH value, the adsorption rate reached 96%. When the pH value was greater than 10.5, the adsorption of U(VI) insignificantly increased. According to the experimental system, Visual MINTEQ 3.1 was used to simulate and analyze the existence form of U(VI) in solution, and the settings of initial conditions were C_[U]_ = 50 mg/L, C_NO3_^−^ = 0.0079 mol L^−1^, C_CO3_^2−^ = 0.001 mol L^−1^, and C_Na+_ = 0.002 mol L^−1^. The morphology distribution of uranium ions in pH 8–14 was simulated, as shown in Figure 5. The decrease in pH is owing to the OH^−^ and CO_3_^−^ adsorption during U(VI) adsorption. Moreover, when pH was 10.5, the main U(VI)-containing ions in the solution are: (UO_2_)_2_CO_3_(OH)_3_^−^, UO_2_(CO)_3_^4−^ and UO_2_(OH)_3_^−^. Therefore, it can be inferred that these three ions are the main forms that are removed by SAIEF [39].Therefore, with the increase of pH, the adsorption rate was obviously higher. The optimal pH in 10.5 was selected for subsequent experiments. Based on the FTIR results, the main ion exchange equation in the range of pH 10–11 can be written as follows:

(3) $$P-{\mathrm{CH}}_{2}N^{+}{\left({\mathrm{CH}}_{3}\right)}_{3}{\mathrm{Cl}}^{-}+{\left({\mathrm{UO}}_{2}\right)}_{2}{\mathrm{CO}}_{3}{\left(\mathrm{OH}\right)}_{3}^{-}\;\to \;P-{\mathrm{CH}}_{2}N^{+}{\left({\mathrm{CH}}_{3}\right)}_{3}{\left({\mathrm{UO}}_{2}\right)}_{2}{\mathrm{CO}}_{3}{\left(\mathrm{OH}\right)}_{3}+{\;\mathrm{Cl}}^{-}$$

(4) $$P-4{\mathrm{CH}}_{2}N^{+}{\left({\mathrm{CH}}_{3}\right)}_{3}{\mathrm{Cl}}^{-}+{\;\mathrm{UO}}_{2}{\left(\mathrm{CO}\right)}_{3}^{4-}\;\to \;P-4{\mathrm{CH}}_{2}N^{+}{\left({\mathrm{CH}}_{3}\right)}_{3}{\left({\mathrm{UO}}_{2}\right)}_{2}{\mathrm{CO}}_{3}^{-}+4{\mathrm{Cl}}^{-}$$

(5) $$P-{\mathrm{CH}}_{2}N^{+}{\left({\mathrm{CH}}_{3}\right)}_{3}{\mathrm{Cl}}^{-}+{\;\mathrm{OU}}_{2}{\left(\mathrm{OH}\right)}_{3}^{-}\;\to P-{\mathrm{CH}}_{2}N^{+}{\left({\mathrm{CH}}_{3}\right)}_{3}{\mathrm{UO}}_{2}{\left(\mathrm{OH}\right)}_{3}+{\;\mathrm{Cl}}^{-}$$

SAIEF is an anion exchange fiber with an anion exchange mechanism for uranium adsorption. The functional group is -CH_2_N + (CH_3_)_3_Cl^−^, where Cl^−^ plays the key role of exchange. Owing to the complex forms of uranium in the solution, the exchange equations of SAIEF uranium removal were obtained by combining environmental water chemical analysis and experimental verification, as shown in Equations (3)–(5).

#### 3.2.2. Effect of Contact Time and Adsorption Kinetics

**Figure 6:**
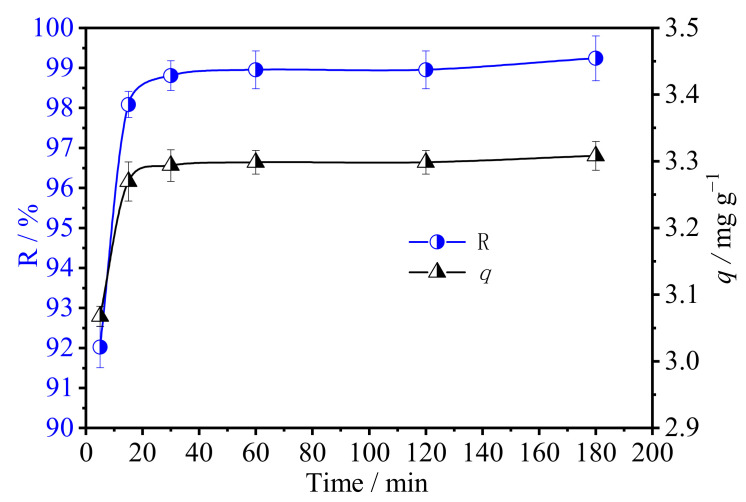
Effect of contact time on the adsorption of U(VI) by SAIEF (C_0_ = 50 mg L^−1^, solid-liquid ratio = 0.15 g L^−1^, pH = 10.5).

To study the equilibrium time, the removal of U(VI) by strong alkaline ion exchange fiber was investigated as a function of contact time under pH of 10.5, C_0_ of 50 mg L^−1^, and solid–liquid ratio of 1.5 g 100 mL^−1^, as shown in Figure 6.

Evidently, U(VI) adsorption quickly attained a steady state and reached adsorption equilibrium at 15–30 min. There are large numbers of unoccupied active sites on modified fibers, which can quickly chelate with U(VI) in solution rapidly, resulting in an obvious increase in the adsorption amount in the first stage. With the adsorption, the active sites on the surface of SAIEF decreased gradually, the electability of the modified fiber also changed, and the repulsive force against U(VI) increased, so that the adsorption reached saturation. 

**Figure 7:**
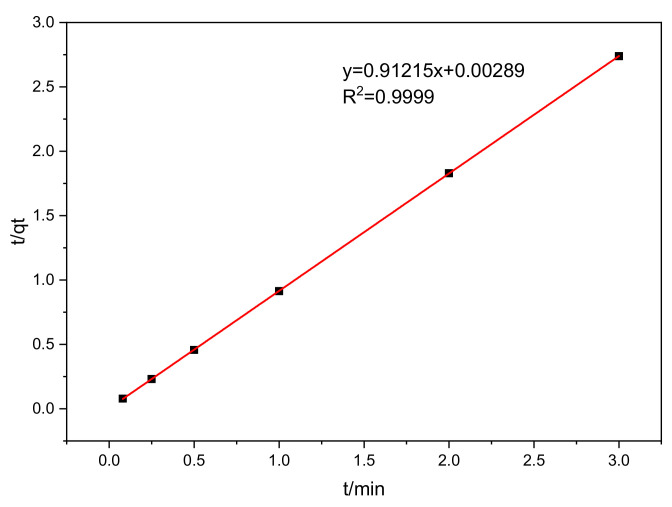
Simulation of kinetics data by pseudo-second-order kinetic model.

To comprehensively understand the effect of contact time, pseudo second-order kinetic models were applied to fit the kinetics data [40]. The parameters are listed in Table 1. The U(VI) adsorption data fitted well with the pseudo second-order kinetic model with a higher correlation coefficient value (R^2^ = 0.9999) (Figure 7). This result indicates that the adsorption process is mainly controlled by the chemical adsorption between functional groups and U(VI). The saturation adsorption of U(VI) was only 3.3 mg L^−1^, due to the low initial concentration of U(VI) and the solution quantity in static experiments (100 mg solution contained only 5 mg U(VI)).

#### 3.2.3. Effect of Initial U(VI) Concentration and Adsorption Isotherm

**Figure 8:**
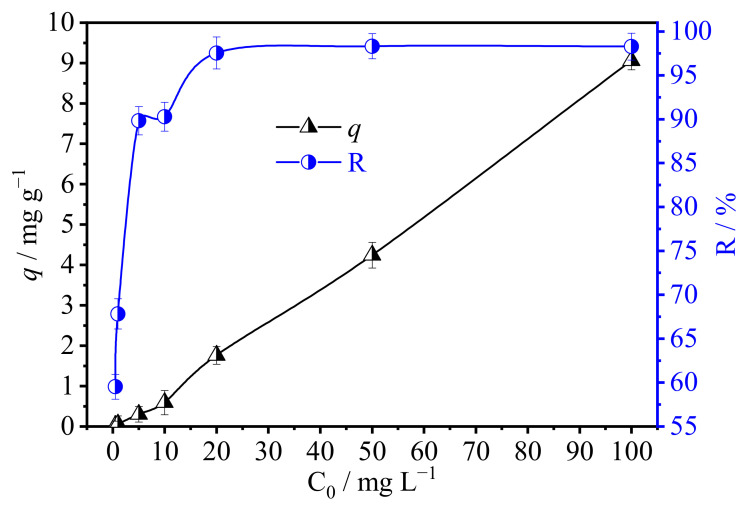
Effect of initial concentration of U(VI) by SAIEF (t = 30 min, m =1 g, pH = 10.5).

To determine the suitable concentration range of U(VI) for SAIEF, the gradient experiments were conducted under the optimal condition of pH of 10.5, contact time of 30 min, and the additional amount of SAIEF of 1 g mL^−1^. As shown in Figure 8, the U(VI) adsorption capacity and rate of SAIEF increased with the increasing initial concentration of U(VI) from 0 to 20 mg/L. When the initial concentration was in the range of 20–100 mg L^−1^, the removal rate of U(VI) by SAIEF remained 97.5% constant, and the most adsorption capacity was approximately 9 mg g^−1^. 

Increasing the initial solution concentration within the appropriate concentration range can increase the collision probability of the modified fiber with U(VI), which could fully utilize the action site on the adsorbent surface and increase the adsorption capacity. When the active adsorption sites on the fiber surface were completely occupied by U(VI), the fiber adsorption reached saturation. Even the initial solution concentration continued to increase, the adsorption capacity remained stable.

To explore U(VI) adsorption behavior by SAIEF, the adsorption isotherm curve of the material was fitted by using Langmuir and Freundlich adsorption isotherm models. Langmuir isothermal equation was originally proposed in the study of gas adsorption. In the application of liquid adsorption, it was assumed that the adsorption was monolayer. The solute and solvent in the adsorption layer were two-dimensional ideal models, implying that each adsorption site can accommodate only one molecule, and the adsorption capacity of each site is the same. The equilibrium adsorption and desorption rate were also similar. The Freundlich adsorption isotherm model is an empirical model. Assuming that the active sites on the adsorbent surface are not uniform and the adsorption is not limited by the monolayer adsorption, the Freundlich adsorption isotherm model can be used to describe reversible adsorption in different systems [41,42,43,44].

As shown in Table 2, Langmuir and Freundlich adsorption isotherm models were used to fit the adsorption data of SAIEF in this study.

**Figure 9:**
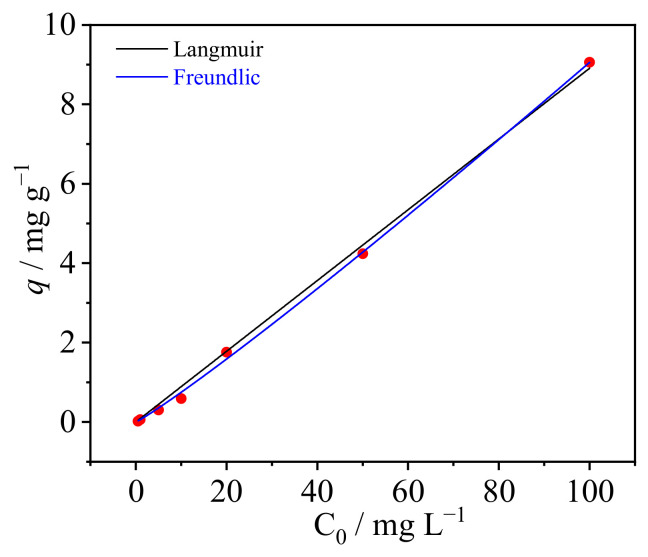
Corresponding experimental data fitted in the Langmuir and Freundlich model.

The associated linear fitting plots are as shown in Figure 9 (Freundlich equation R^2^ = 0.8273 and Langmuir equation R^2^ = 0.8496), indicating that the Langmuir adsorption isothermal equation described the adsorption process of uranium in solution better than the Freundlich adsorption isothermal equation [45]. The results reveal that the ion exchange process of ion exchange fibers is monolayer adsorption. In the Freundlich model, when the adsorption process was difficult, the equilibrium constant n is smaller than 0.5, and when the adsorption was easy to perform, the constant n was in the range of 2–10 [46]. The result, that equilibrium constant (n) of SAIEF is 2.7697, demonstrates that SAIEF material has excellent adsorption potential for U(VI) removal from wastewater. The high capacity of SAIEF may be because of the large number of chloride ions on the adsorbent. More precisely, because there are many unoccupied and available active sites on the SAIEF surface in the initial phase, the adsorption increased rapidly. Finally, the trend slowed down at high concentrations.

#### 3.2.4. Effect of the Fiber Dosage

To explore the effect of fiber amount on adsorption, 0.5 g, 1 g, 5 g, and 2 g of ion exchange fiber were added in 100 mL U(VI) −solution at room temperature (25 °C), respectively. The initial concentration of U(VI) solution was 50 mg L^−1^, pH was 10.5, and contact time was 2 h. 

**Figure 10:**
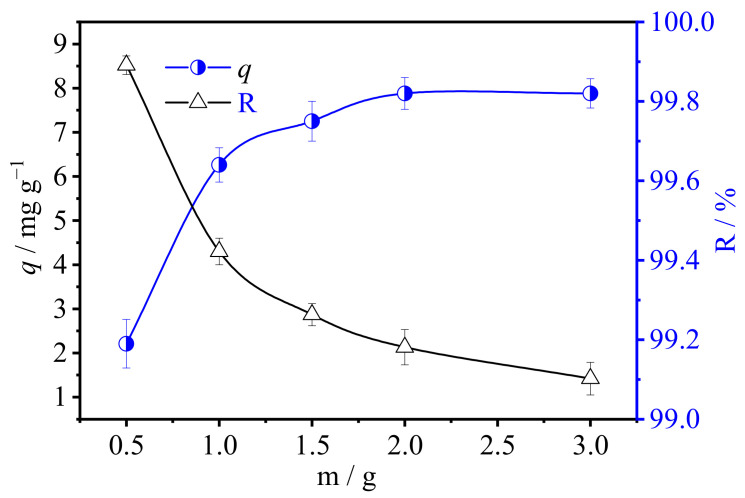
Effect of SAIEF dosage on the adsorption of U(VI) (t = 120 min, C_0_ = 50 mg L^−1^, pH = 10.5).

As shown in Figure 10, although the adsorption capacity decreased with the increase in fiber dosage, indicating that, before ion exchange fiber reaches saturation adsorption capacity, the higher the dosage is, the better the adsorption effect will be. The possible reason is, more SAIEF can provide more adsorption sites and increase the effective contact time of ion exchange adsorption. U(VI)-containing wastewater can fully contact SAIEF, thus accelerating the adsorption of uranium complex ions [47]. However, considering the influence of economic factors, the fiber dosage should be adjusted appropriately. In subsequent experiments, the fiber dosage was selected as 1.5 g 100 mL^−1^. The adsorption rate of SAIEF then reached 99.7% for the low-concentration U(VI) solution, implying that high U(VI) concentrations in wastewater could be removed. The study demonstrates that SAIEF has excellent adsorption potential for the extraction of U(VI) removal from an aqueous medium.

### 3.3. Gradient Descent Experiments with Constant Solid-to-Liquid Ratio

**Figure 11:**
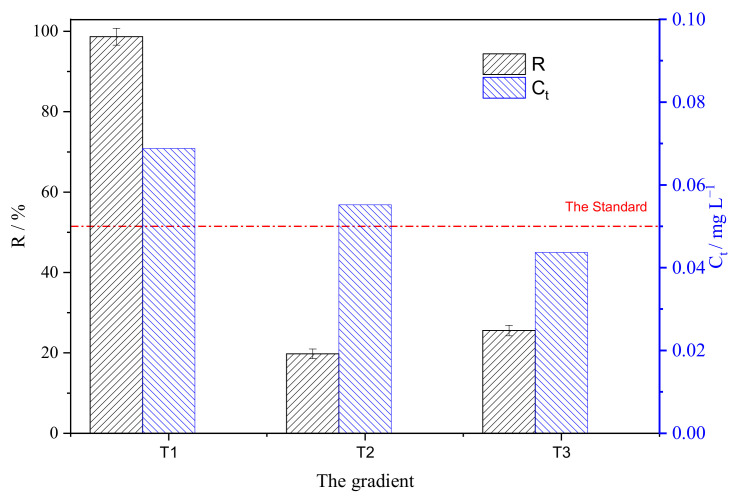
SAIEF solid-liquid ratio conditions of uranium concentration gradient descent results.

To simulate experiments of three columns in series and clarify the ability of SAIEF for deep purification of U(VI) wastewater, the removal of U(VI) by SAIEF was studied as a function of gradient experiments with constant solid-to-liquid ratio (1.5 g 100 mL^−1^), as shown in Figure 11. Considering the errors due to sampling, each fiber dosage of fibers decreased in proportion.

After three times adsorption cycles under constant solid–liquid ratio, the uranium concentration has been reduced from 50 mg L^−1^ to 0.0437 mg L^−1^, achieving the GB23727-2009 national standard value for the direct discharge of uranium concentration (0.05 mg L^−1^) in China. The results also show that the strong alkali ion exchange fiber used in this study has the ability of adsorption recovery and deep purification of uranium with low concentration. The follow-up dynamic test could use the 2–3 cascades.

### 3.4. Dynamic Adsorption

#### 3.4.1. Saturated Adsorption Capacity Determination

**Figure 12:**
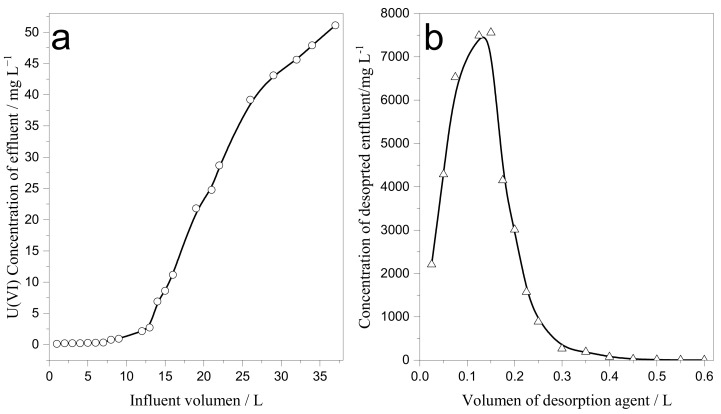
Uranium initial concentration penetration test at 50 mg L^−1^ ((**a**): breaking through curve; (**b**): desorption curve).

The saturated adsorption capacity determination was studied in a column of 3 cm size by packing 3.0 g of SAIEF. The length of the column was 3 cm. The U(VI) solution (C_0_ = 50 mg/L) was passed through the column at a flow rate of 15–20 mL min^−1^. Subsequently, 0.1 mol L^−1^ HCL was used for the desorption of fiber at a flow rate of 7 mL/min. The penetration and desorption curves of the results are shown in Figure 12. Evidently, saturated sorption capacity of the fiber for uranium was 423.9 mg g^−1^, and the desorption rate reached 99.5%, indicating the excellent adsorption performance for U(VI) ions and reusability of the adsorbent.

Comparing the adsorption capacity of the SAIEF against other adsorbents (in Table 3), it is obvious that this fiber has good potential for uranium removal, owing to the fibrous structure, SAIEF, with larger specific surface area and less resistance to water flow.

#### 3.4.2. Two-Grade Serial Dynamic Adsorption Experiment

**Figure 13:**
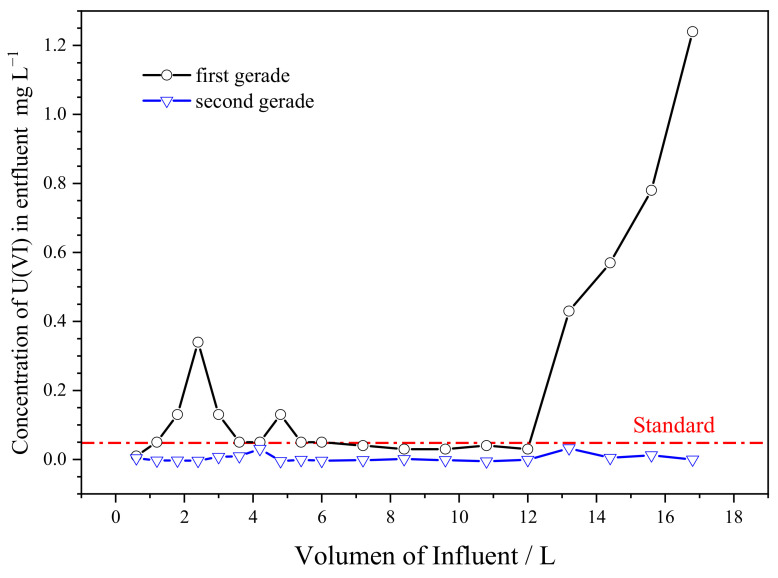
Two-grade serial effluent concentration curve.

With the same conditions (pH, flow rate and initial concentration of U(VI)), the dynamic adsorption ability of SAIEF was also studied through a two-grade serial ion exchange fiber column, with each column packed with 20 g fibers (diameter of 3.5 cm; high of 20 cm; and density of 0.103 g cm^−3^). As shown in Figure 13, the effluent U(VI) concentration of the first-stage ion exchange column was maintained at 0.05–0.10 mg /L^−1^, which failed to meet the national environmental discharge standard (GB23727-2009). Due to the existence of grooves, the wastewater could not achieve full contact with the fibers. Part of the wastewater with high U(VI) concentration flew out through the grooves. When the adsorption capacity of fiber reached 30 mg g^−1^, the fiber’s adsorption capacity for low-concentration uranium decreased, and the effluent U(VI) concentration consequently increased.

**Figure 14:**
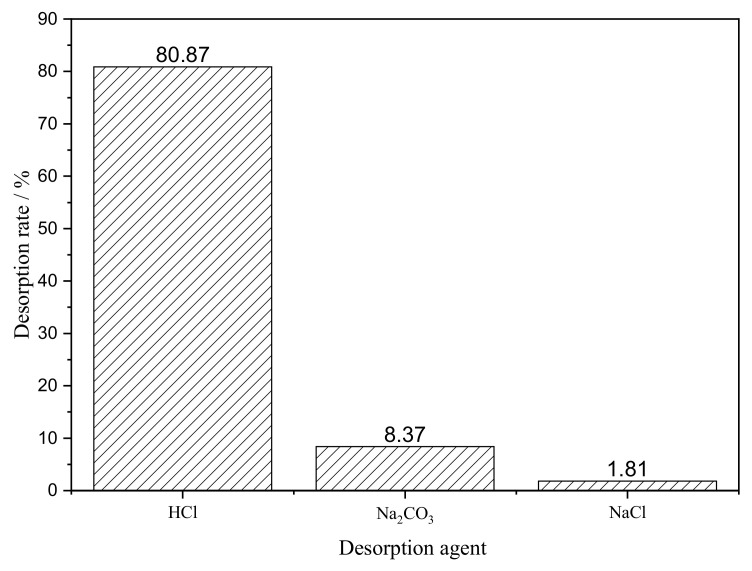
Screening of desorption agent for SAIEF.

The effluent U(VI) concentration of the second-stage ion exchange column was maintained at 0.01–0.045 mg L^−1^, meeting the requirements of depth purification. Moreover, because of the existence of grooves, the concentration curve in Figure 14 was not completely smooth, and had slight undulation. It is recommended to use a counter-current column to reduce the influence of the groove, and design 2–4 stages in series depending on the concentration of uranium in the influent.

### 3.5. Repeatability

Under the optimal adsorption test conditions, this study compared the static desorption effect of three kinds of 0.1 mol L^−1^ desorption solutions on uranium-removal by fiber, as shown in Figure 14. The desorption effect of 0.1 mol L^−1^ HCl was the best, reaching 80.87%.

**Figure 15:**
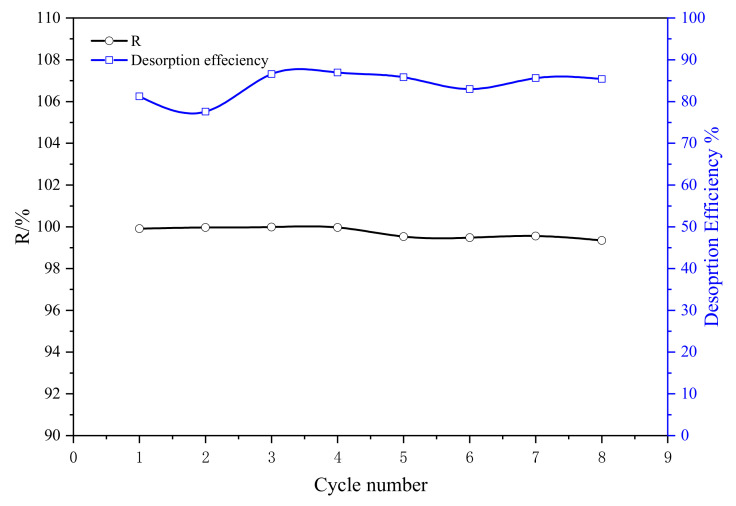
HCl desorption and reabsorption test results (SAIEF).

As shown in Figure 15, after the eighth successive adsorption cycle, the U(VI) removal efficiency of SAIEF was 99%. The result indicated that, 0.1 mol L^−1^ HCl was feasible as desorption agent and the fiber was excellently reusable.

## 4. Conclusions

Strong alkaline ion exchange fiber (SAIEF) is a novel type of ion exchange fiber with high adsorption capacity. According to the experiments, when the fiber dosage was 1.5 g 100 mL^−1^ and the pH was 10.5, the uranium removal efficiency was optimal, and the removal efficiency reached more than 99% in the U(VI) concentration range of 20–40 mg L^−1^ Repeated adsorption/desorption experiments revealed that the SAIEF has characteristics of regeneration and reusability to uranium removal, and the optimal desorption agent is dilute HCl. The adsorption mechanism of uranium by SAIEF is anion exchange, and the functional group is CH_2_N^+^(CH_3_)_3_Cl^−^, where Cl^−^ plays a crucial role in adsorption. In addition, dynamic experiments reveal that SAIEF possesses a remarkable ability for deep uranium purification. The results suggest that strong alkaline ion exchange fiber (SAIEF) is an excellent material for uranium removal.

## Figures and Tables

**Table 1:** Parameters of pseudo second order kinetic model.

K_2_ [g (mg·min)^−1^]	q_e_ (mg g^−1^)	R^2^
287.9011	1.0963	0.9999

**Table 2:** Adsorption isotherm parameters of SAIEF.

	n	K_f_/(mg·g^−1^)	k_a_/(L·mg^−1^)	q_m_/(mg·g^−1^)	R^2^
Langmuir			1.4738	0.0819	0.8496
Freundlich	2.7697	4.3647			0.8273

**Table 3:** Comparison of adsorption capacity of various adsorbents for uranium from water.

Material	Time (h)	q_e_ (mg g^−1^)	Reference
SA/CMC-Ca-Al	2	101.76	[10]
PAMAM	3	215.5	[48]
PAN-P4-A6	0.67	200.1	[49]
SAIEF	0.5	423.9	this study

## Data Availability

Not applicable.

## References

[1] Li J., Wang X., Zhao G., Chen C., Chai Z., Alsaedi A., Hayatf T., Wang X. (2018). Metal-organic framework-based materials: Superior adsorbents for the capture of toxic and radioactive metal ions. Chem. Soc. Rev..

[2] Uggla Y. (2004). Risk and safety analysis in longterm perspective. Futures.

[3] Hogan A.C., VanDam R.A., Markich S., Camilleri C. (2005). Chronic toxicity of Uranium to a tropical green alga (Chlorellasp.) in natural waters and the influence of dissolved organic carbon. Aquat. Toxicol..

[4] Vigier J.-F., Laplace A., Renard C., Miguirditchian M., Abraham F. (2018). Uranium (III)-Plutonium (III) co-precipitation in molten chloride. J. Nucl. Mater..

[5] Zhou Q., Tan K., Zeng S., Liu D. (2009). Synergetic treatment of Urannium-Bearing Wstewater with Sulfate Reducing Bcteria and Zero-Valent Iron. At. Energy Sci. Technol..

[6] Crane R.A., Dickinson M., Popescu I.C., Scott T.B. (2011). Magnetite and zero-valent iron nanoparticles for the remediation of uranium contaminated environmental water. Water Res..

[7] Noubactep C., Schoner A., Meinrath G. (2006). Mechanism of Uranium removal from the aqueous solution by elemental iron. J. Hazard. Mater..

[8] Ding C.C., Cheng W.C., Nie X.Q., Yi F.C. (2017). Synergistic mechanism of U(VI) sequestration by magnetite-graphene oxide composites: Evidence from spectroscopic and theoretical calculation. Chem. Eng. J..

[9] Mar Camacho L., Deng S., Parra R.R. (2010). Uranium removal from groundwater by natural clinoptilolite zeolite: Effects of pH and initial feed concentration. J. Hazard. Mater..

[10] Wu L., Lin X., Zhou X., Luo X. (2016). Removal of uranium and fluorine from wastewater by double-functional microsphere adsorbent of SA/CMC loaded with calcium and aluminum. Appl. Surf. Sci..

[11] Kumar S., Loganathan V.A., Gupta R.B., Barnett M.O. (2011). An Assessment of U (VI) removal from groundwater using biochar produced from hydrothermal carbonization. J. Environ. Manag..

[12] Wang G., Liu J., Wang X., Xie Z., Deng N. (2009). Adsorption of Uranium (VI) from aqueous solution onto cross-linked chitosan. J. Hazard. Mater..

[13] Bai J., Yao H., Fan F., Lin M., Zhang L., Ding H., Lei F., Wu X., Li X., Guo J. (2010). Biosorption of Uranium by chemically modified Rhodotorula glutinis. J. Environ. Radioact..

[14] Liang P.-l., Yuan L.-y., Deng H., Wang X.-c., Wang L., Li Z.-j., Luo S.-z., Shi W.-q. (2020). Photocatalytic reduction of uranium(VI) by magnetic ZnFe2O4. Appl. Catal., B..

[15] Theerthagiri J., Lee S.J., Karuppasamy K., Arulmani S., Veeralakshmi S., Ashokkumar M., Choi M.Y. (2021). Application of advanced materials in sonophotocatalytic processes for the remediation of environmental pollutants. J. Hazard. Mater..

[16] Malovanyy A., Plaza E., Trela J., Malovanyy M. (2014). Combination of ion exchange and partial nitritation/Anammox process for ammonium removal from mainstream municipal wastewater. Water Sci. Technol..

[17] Wang P., Lv C.-X., Sheng Q., Sun H.-T., Zhang L. (2016). Research development of uranium-containing wastewater treatment technologies. Xiandai Huagong/Mod. Chem. Ind..

[18] Chen T., Zhang J., Ge H., Li M., Li Y., Liu B., Duan T., He R., Zhu W. (2020). Efficient extraction of uranium in organics-containing wastewater over g-C 3 N 4 /GO hybrid nanosheets with type-II band structure. J. Hazard. Mater..

[19] Atia B.M., Sakr A.K., Gado M.A., El-Gendy H.S., Abdelazeem N.M., El-Sheikh E.M., Hanfi M.Y., Sayyed M.I., Al-Otaibi J.S., Cheira M.F. (2022). Synthesis of a New Chelating Iminophosphorane Derivative (Phosphazene) for U(VI) Recovery. Polymers.

[20] Koid Y., Uchino M., Yamada K. (2006). Studies of collectors. ix. the flotation of a trace amount of uranium by using 2-(alkylamino)propionohydroxamic acid and cotelomer-type surfactants bearing hydroxyaminocarbonyl and pyridyl groups. Bull. Chem. Soc. Jpn..

[21] Yarlagadda S., Gude V.G., Camacho L.M., Pinappu S., Deng S. (2011). Potable water recovery from As, U, and F contaminated ground waters by direct contact membrane distillation process. J. Hazard. Mater..

[22] Molinari R., Argurio P., Poerio T. (2009). Membrane Processes Based on Complexation Reactions of Pollutants as Sustainable Wastewater Treatments. Sustainability.

[23] Guruprashanth N., Hegde R., Suresh B. (2021). A Review on Organic Adsorbents for the Removal of Toxic Metals from Waste Water. Asian J. Adv. Res. Rep..

[24] Sakr A.K., Al-Hamarneh I.F., Gomaa H., Abdel Aal M.M., Hanfi M.Y., Sayyed M.I., Khandaler M.U., Cheira M.F. (2022). Removal of uranium from nuclear effluent using regenerated bleaching earth steeped in β-naphthol. Radiat. Phys. Chem..

[25] Li L., Li H., Lin M., Wen J., Hu S. (2023). Effects of chain conformation on uranium adsorption performance of amidoxime adsorbents. Sep. Purif. Technol..

[26] Huang G., Li W., Liu Q., Liu J., Zhang H., Li R., Li Z., Jing X., Wang J. (2018). Effecient removal of uranium(VI) from simulated seawater with hyperbranchedpolyethylenimine(HPEI)—functionalized polyacrylonitrile fibers. New J. Chem..

[27] Yin L., Hu B., Zhuang L., Fu D., Li J., Hayat T., Alsaedi A., Wang X. (2020). Synthesis of flexible cross-linked cryptomelane-type manganese oxide nanowire membranes and their application for U(VI) and Eu(III) elimination from solutions. Chem. Eng. J..

[28] Xia X., Dong F., Nie X., Pan N., Liu C., Ding C., Wang J., Cheng W., He H., Sun S. (2022). Efficient adsorption of U(VI) using in low-level radioactive wastewater containing organic matter by amino groups modified polyacrylonitrile fibers. J. Radioanal. Nucl. Chem..

[29] Nie X., Zhang Y., Jiang Y., Pan N., Liu C., Wang J., Ma C., Xia X., Liu M., Zhang H. (2022). Efficient extraction of u(vi) from uranium enrichment process wastewater by amine-aminophosphonate-modified polyacrylonitrile fibers. Sci. Total Environ..

[30] Zhou C., Li M., Zeng Q. (2003). Study on adsorption of heavy mental by ion exchange fiber. Tech. Equip. Environ. Pollut. Control.

[31] Zeng H., Yu W. (1987). Study on adsorption behavior of heavy metal ions by new cation exchange fibers. Technol. Water Treat..

[32] Zheng X., Ye L., Jiang J., Wu X., Wu W. (2019). Determination of lead and cadmium in regenerated zinc by inductively coupled plasma atomic emission spectrometry after extraction separation with strongly basic anion exchange fiber. Met. Anal..

[33] Mihrin D., Andersen J., Jakobsen P.W., Larsen R.W. (2019). Highly localized H2O librational motion as a far-infrared spectroscopic probe for microsolvation of organic molecules. Phys. Chem. Chem. Phys..

[34] Jiang Y.H., Sun C.L., Li Z.L., Cao A.Y., Li Z.W. (2011). Enhanced stimulated Raman scattering of weak-gain mode C-H stretching vibration of benzene. Acta Phys. Sin. (Overseas Ed.).

[35] Zhao D., Wang Z., Lu S., Shi X. (2020). An amidoxime-functionalized polypropylene fiber: Competitive removal of Cu(II), Pb(II) and Zn(II) from wastewater and subsequent sequestration in cement mortar. J. Clean. Prod..

[36] Li Y., Simon A.O., Jiao C., Zhang M., Yan W., Rao H., Liu J., Zhang J. (2020). Rapid removal of Sr2+, Cs+ and UO22+ from solution with surfactant and amino acid modified zeolite Y. Microporous Mesoporous Mater..

[37] Ahmed W., Nunez-Delgado A., Mehmood S., Ali S., Qaswar M., Shakoor A., Chen D. (2021). Highly efficient uranium (VI) capture from aqueous solution by means of a hydroxyapatite-biochar nanocomposite: Adsorption behavior and mechanism. Environ. Res..

[38] Su S., Chen R., Liu Q., Liu J., Zhang H., Li R., Zhang M., Liu P., Wang J. (2018). High efficiency extraction of U(VI) from seawater by incorporation of polyethyleneimine, polyacrylic acid hydrogel and luffa cylindrical fibers. Chem. Eng. J..

[39] Zhang J., Luo X. (2021). Bioaccumulation characteristics and acute toxicity of uranium in Hydrodictyon reticulatum: An algae with potential for wastewater remediation. Chemosphere.

[40] El-Shahawi M., El-Sonbati M. (2005). Retention profile, kinetics and sequential determination of selenium(IV) and (VI) employing 4,4′-dichlorodithizone immobilizedpolyurethane foams. Talanta.

[41] Kavitha D., Namasivayam C. (2007). Experimental and kinetic studies on methylene blue adsorption by coir pith carbon. Bioresour. Technol..

[42] Zhu K., Gao Y., Tan X., Chen C. (2016). Polyaniline-modified Mg/Al layered double hydroxide composites and their application in efficient removal of Cr(VI). ACS Sustain. Chem. Eng..

[43] Chen Z., Chen W., Jia D., Liu Y., Zhang A., Wen T., Liu J., Ai Y., Song W., Wang X. (2018). N, P, and S codoped graphene-Like carbon nanosheets for ultrafast uranium(VI) capture with high capacity. Adv. Sci..

[44] Deng S., Wang P., Zhang G., Dou Y. (2016). Polyacrylonitrile-based fiber modified with thiosemicarbazide by microwave irradiation and its adsorption behavior for Cd(II) and Pb(II). J. Hazard. Mater..

[45] Kumar P.A., Ray M., Chakraborty S. (2009). Adsorption behaviour of trivalent chromium on amine-based polymer aniline formaldehyde condensate. Chem. Eng. J..

[46] Liu X., Li J., Wang X., Chen C., Wang X. (2015). High performance of phosphatefunctionalized graphene oxide for the selective adsorption of U(VI) from acidic solution. J. Nucl. Mater..

[47] Guo Y., Huang T., Wen G., Cao X. (2017). The simultaneous removal of ammonium and manganese from groundwater by iron-manganese co-oxide filter film: The role of chemical catalytic oxidation for ammonium removal. Chem. Eng. J..

[48] Wang F., Liao Y., Xia L. (2021). Poly(amidoamine) dendrimer decorated dendritic fibrousnano-silica for efficient removal of uranium (VI). J. Solid State Chem..

[49] Cheng Y., He P., Dong F., Nie X., Ding C., Wang S., Zhang Y., Liu H., Zhou S. (2019). Polyamine and amidoxime groups modified bifunctional polyacrylonitrile-based ion exchange fibers for highly efficient extraction of U(VI) from real uranium mine water. Chem. Eng. J..

